# Supplementary material for: Deciphering local and regional hydroclimate resolves contradicting evidence on the Asian monsoon evolution
Source: Nat Commun. 2023 Sep 14;14:5697. doi: 10.1038/s41467-023-41373-9 (PMC10502020; doi:10.1038/s41467-023-41373-9)
Supplement: Supplementary file 1 — Supplementary Information [file 41467_2023_41373_MOESM1_ESM.pdf]

Supplementary Materials for

Deciphering local and regional hydroclimate resolves contradicting evidence on the Asian monsoon evolution

Annabel Wolf*, Vasile Ersek*, Tobias Braun, Amanda D. French, David McGee, Stefano M. Bernasconi, Vanessa Skiba, Michael L. Griffiths, Kathleen R. Johnson, Jens Fohlmeister, Sebastian F. M. Breitenbach, Francesco S. R. Pausata, Clay Tabor, Jack Longman, William H. G. Roberts, Deepak Chandan, W. Richard Peltier, Ulrich Salzmann, Deborah Limbert, Hong Quan Trinh, Anh Duc Trinh

*Corresponding author. Email: wolfa2@uci.edu and vasile.ersek@northumbria.ac.uk

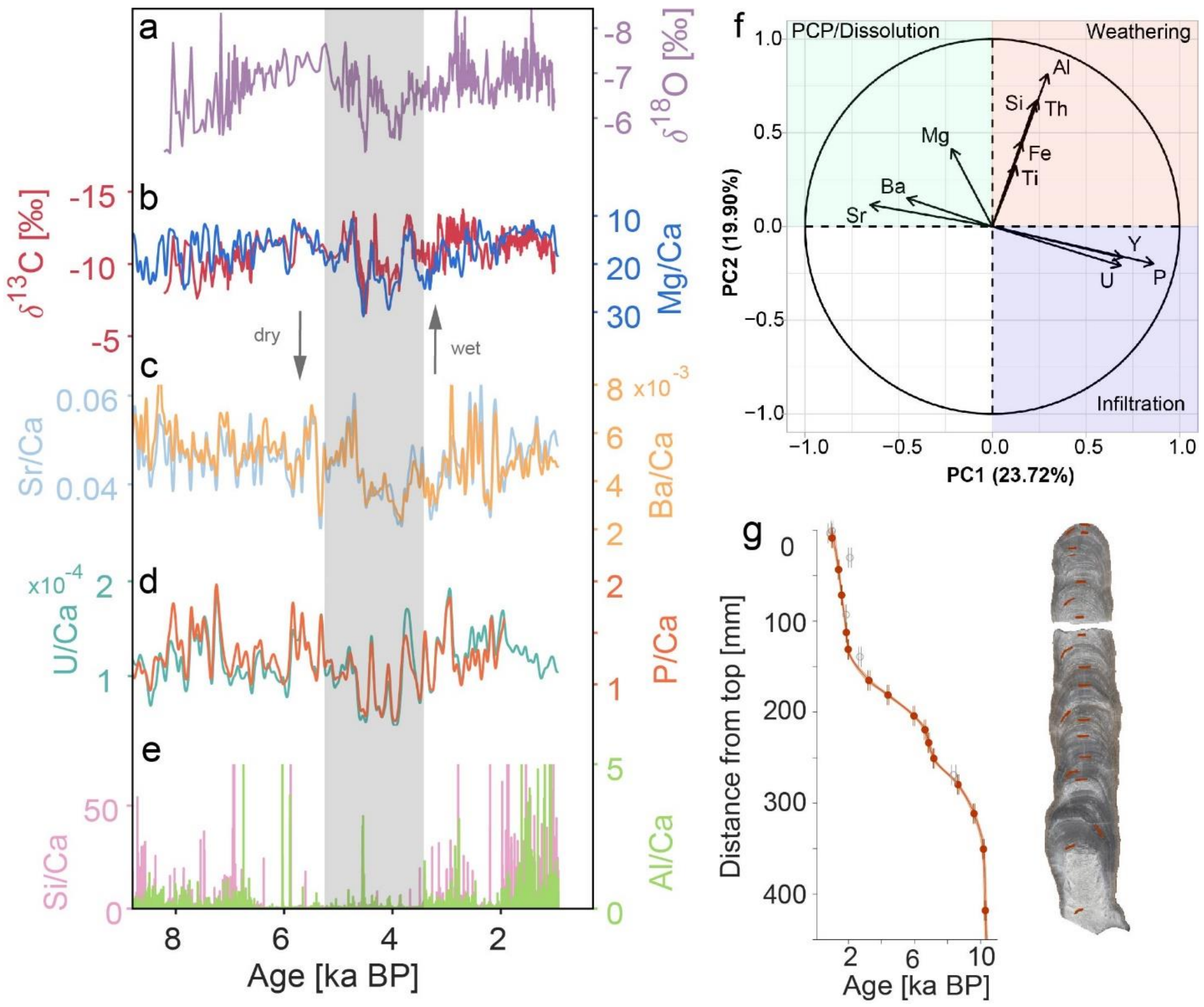

Fig. S1. Multi-proxy record, PCA of trace elements and age-depth model of TD3.

(**a**) TD3 δ^{18}O reflects changes in moisture source dynamics, (**b**) TD3 δ^{13}C is superimposed with a smoothed time series of Mg/Ca, and (**c**) Sr/Ca and Ba/Ca, recording local hydrological conditions, (**d**) smoothed U/Ca and P/Ca ratios indicate short-term infiltration changes. (**e**) TD3 Si/Ca and Al/Ca ratios are interpreted as proxies for increased weathering. **(f)** PCA loadings of elements typical for PCP (Sr, Ba, Mg), weathering (Al, Si, Fe, Ti, Th) and infiltration (U, P,Y) in stalagmite TD3. **(g)** Age model and scan of stalagmite TD3 with red marks showing the location of samples for U/Th dating. Delta values given in VPDB and trace element ratios as mmol/mol.

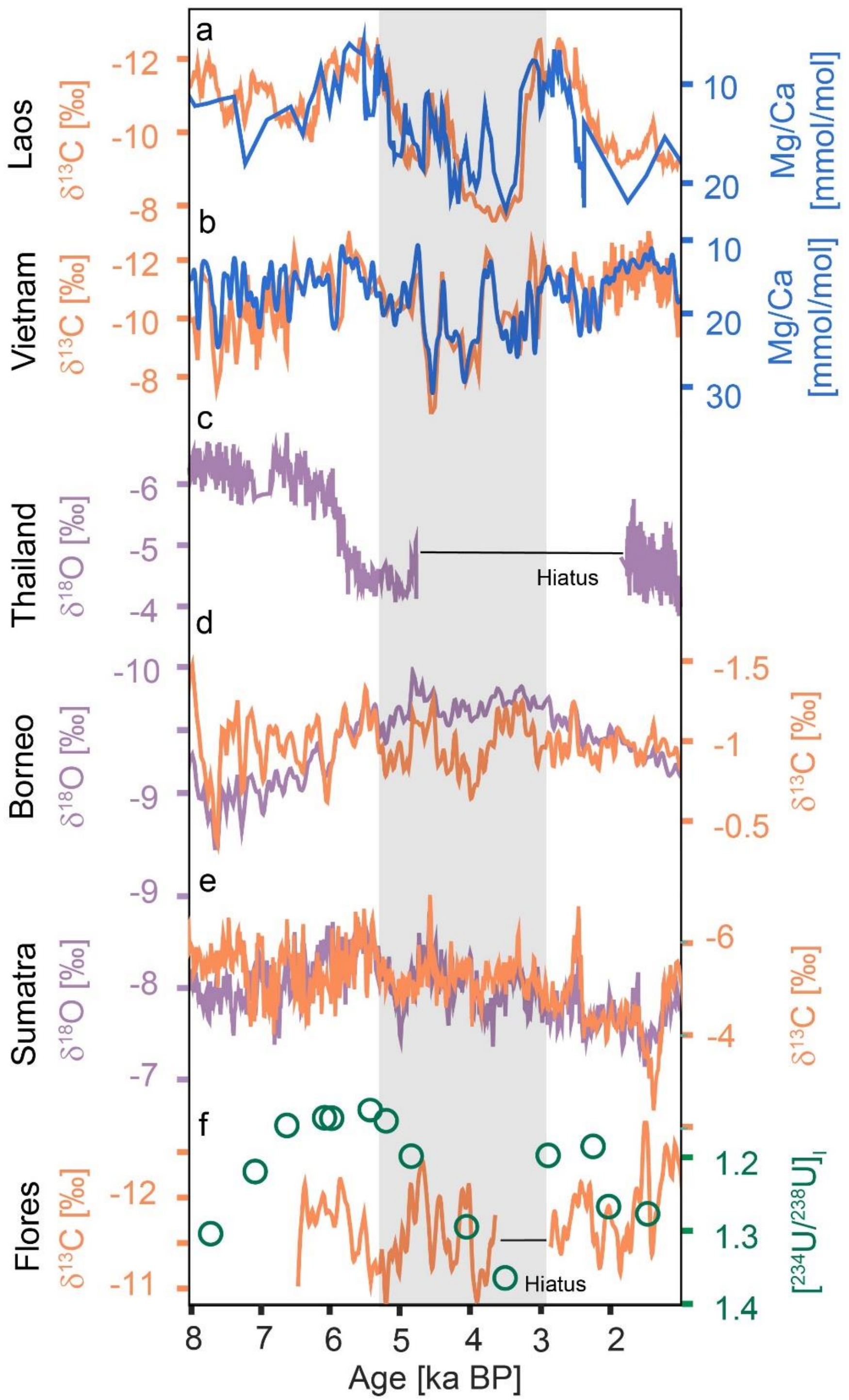

Fig. S2. Speleothem-based monsoon proxies from across Southeast Asia.

(a) δ^{13}C and Mg/Ca record from Tham Mai Cave, Laos[1] **(b)** δ^{13}C and Mg/Ca record of TD3, **(c)** δ^{18}O record from Klang Cave, southern Thailand[2] **(d)** δ^{18}O and δ^{13}C from Bukit Assam Cave, Borneo[3], **(e)** δ^{18}O and δ^{13}C record from Tangga Cave, Sumatra[4]. **(f)** δ^{13}C and initial U record from Liang Luar Cave, Flores[5]. Grey shading indicates the extreme dry conditions in parts of Southeast Asia during the Holocene. Delta values given as VPDB.

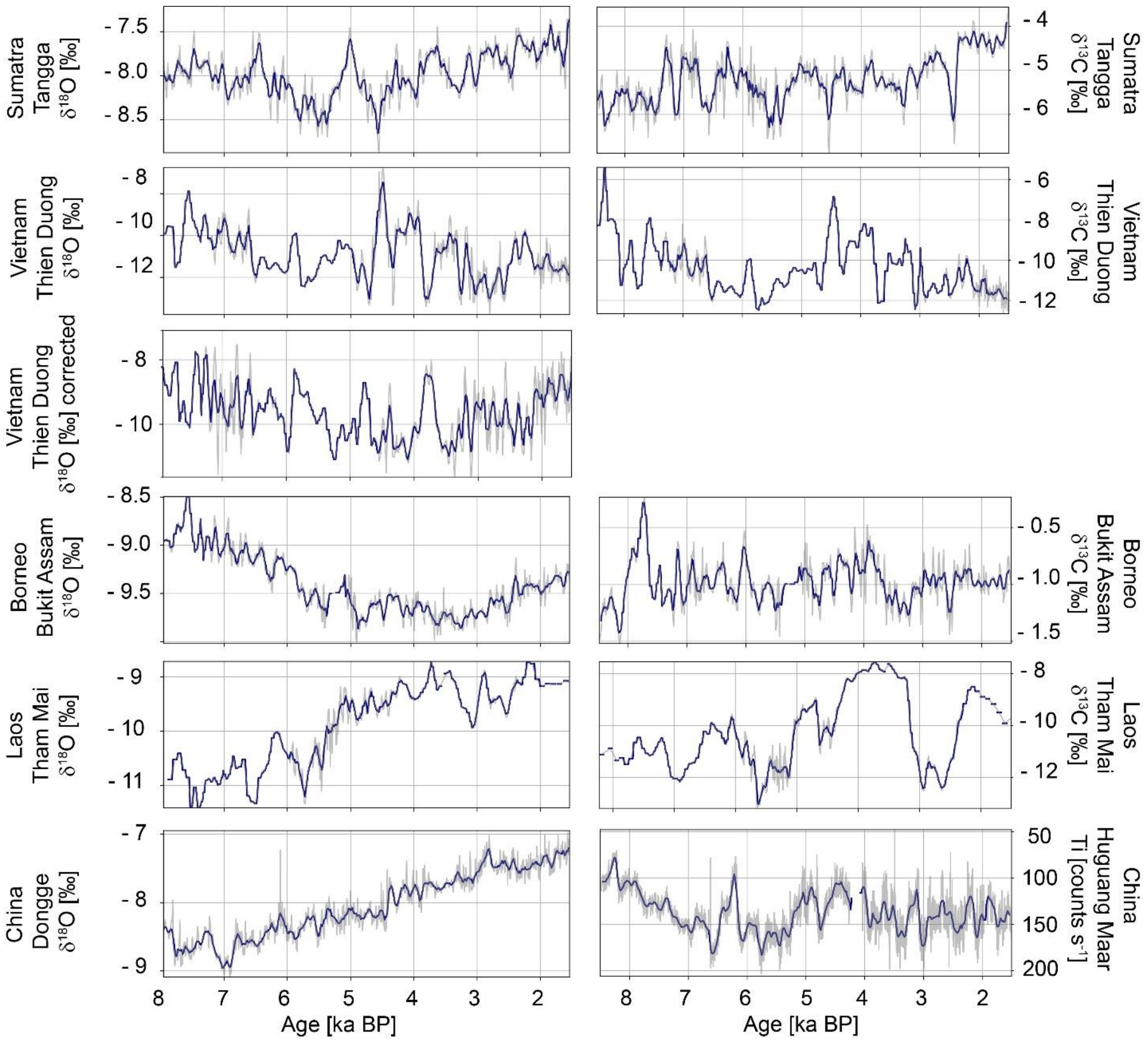

Fig. S3. Speleothem-based monsoon proxies used for the MC-PCA.

Left panel shows oxygen isotope records and the right panel shows carbon isotopes records, except for the Huguang Maar record which shows the titanium content[6].

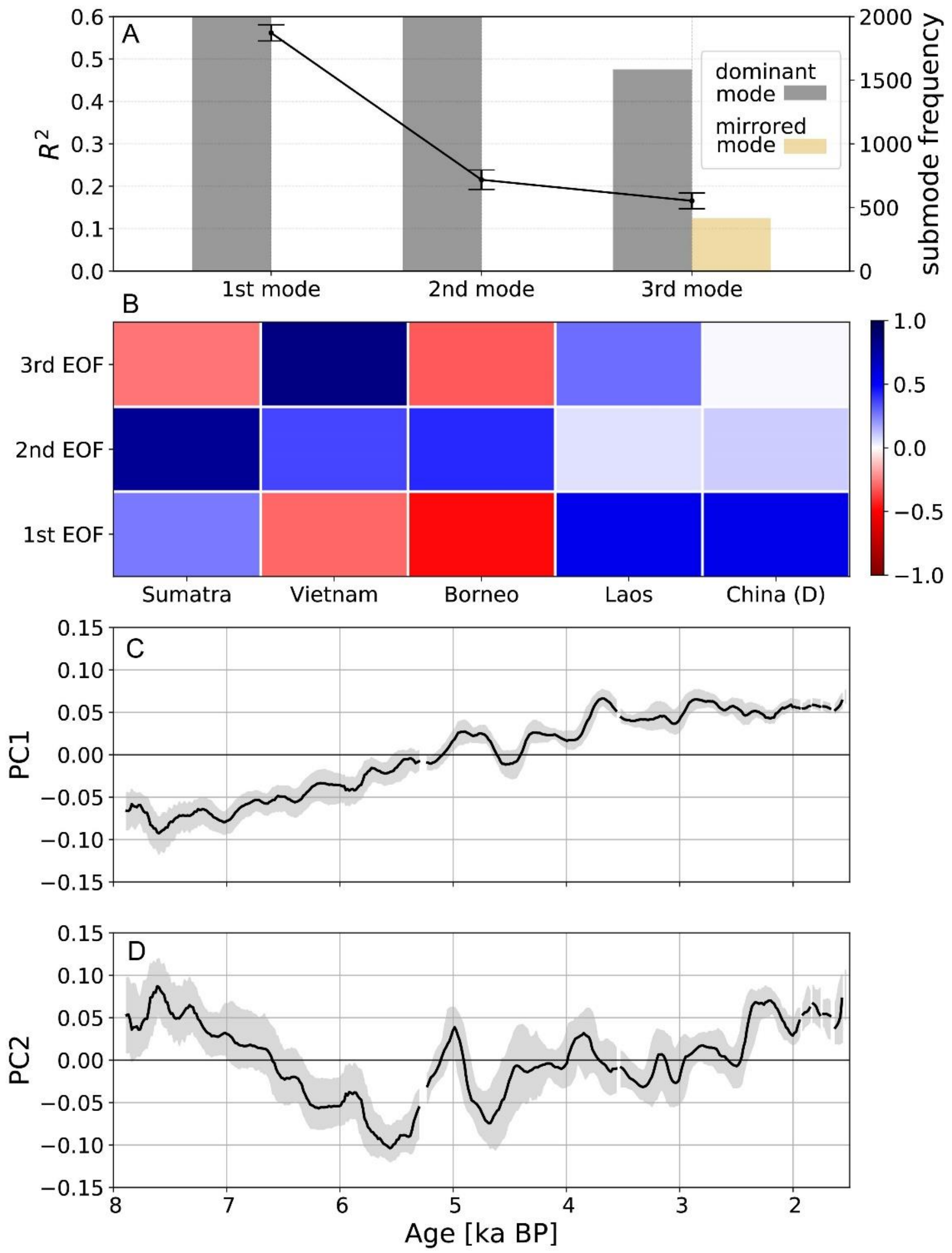

Fig. S4. MC-PCA of *original* δ^{18}O records.

(a) mean R^2 indicates the variance explained by each mode (dashed lines), with 2σ-errorbars for different R^2 estimates, resulting from the different age realizations. Due to the clustering there is a dominant mode (grey bar), which uses most of the age model realisations and a mirrored mode describing the residuals (yellow bar). (**b**) Averaged EOF for each mode and each ensemble of proxy time series. The EOF gives a value of correlation between proxy time series and the individual PC. First (**c**) and second **(d)** leading PCs extracted from the five δ^{18}O records. The solid line shows the dominant PC, dashed yellow line shows the sign-flipped PC (after flipping, only in **(d)**) and the shading represent the 2σ error region propagated from the dating uncertainties for (**c**) PC1 and **(d)** PC2.

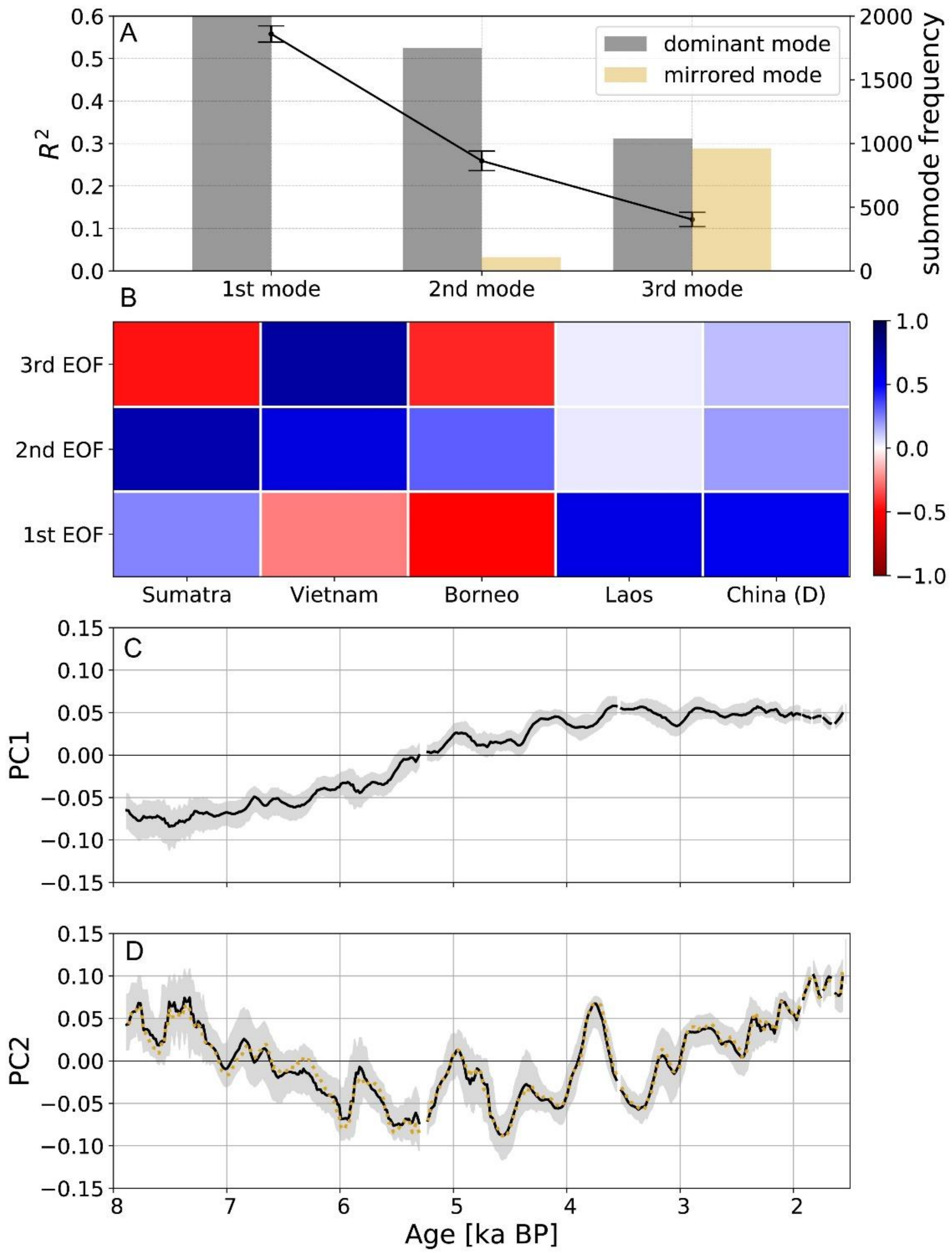

Fig. S5. MC-PCA δ^{18}O records with TD3 *corrected* for PCP.

(a) Mean R^2 indicates the variance explained by each mode (dashed lines), with 2σ-errorbars for different R^2 estimates, resulting from the different age realizations. Due to the clustering there is a dominant mode (grey bar), which uses most of the age model realisations and a mirrored mode describing the residuals (yellow bars). (**b**) Averaged EOF for each mode and each ensemble of proxy time series. The EOF gives a value of correlation between proxy time series and the individual PC. First (**c**) and second **(d)** leading PCs extracted from the five δ^{18}O records and the TD3 has been corrected for PCP. The solid line shows the dominant PC, dashed yellow line shows the sign-flipped PC (after flipping, only in **(d)**) and the shading represents the 2σ error region propagated from the dating uncertainties for (**c**) PC1 and **(d)** PC2.

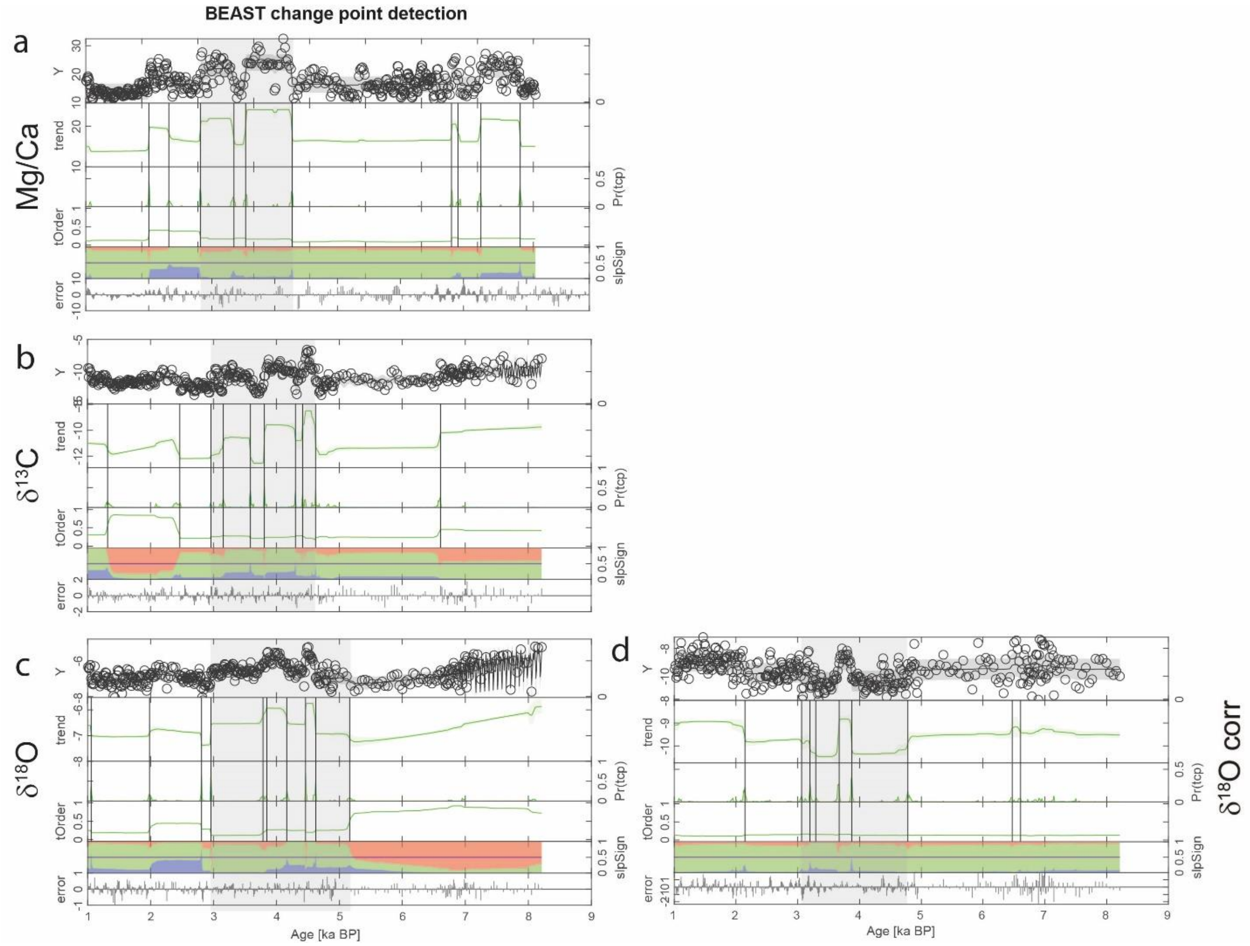

Fig. S6. Change point detection for the proxy record of TD3.

BEAST change point detection for (a) Mg/Ca, (b) δ^{13}C, (c) δ^{18}O original and (d) corrected. Top plots (Y) in each panel show the data points plotted as circles and the mean as line plot. The trend is also plotted in the second panel from the top for better visibility. Third line plot (Pr(tcp)) show the probability of changepoint occurrences over time. "tOrder" shows the time-varying polynomial order estimated to fit trend (close to zero, meaning a flat /constant line). "slpSign" is the probabilities of trend slope being positive (red), zero (green), or negative (blue).

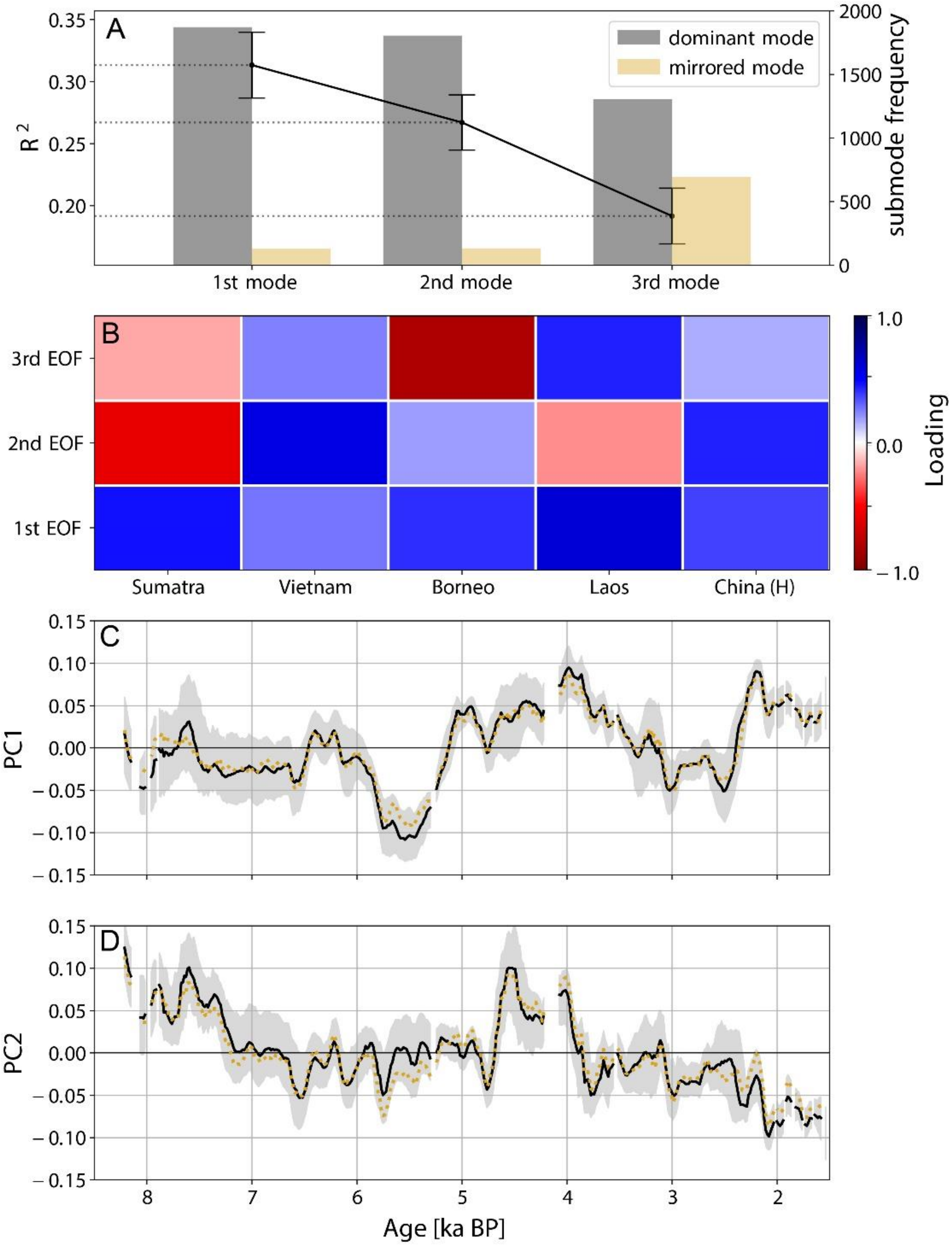

Fig. S7. MC-PCA of the δ^{13}C records.

(a) mean R^2 indicates the variance explained by each mode (dashed lines), with 2σ-errorbars for different R^2 estimates, resulting from the different age realizations. Due to the clustering there is a dominant mode (grey bar), which uses most of the age model realisations and a mirrored mode describing the residuals (yellow bar). (**b**) Averaged EOF for each mode and each ensemble of proxy time series. The EOF gives a value of correlation between proxy time series and the individual PC. (**c**)/(**d**) Two leading PCs extracted from the five δ^{13}C records. The solid line shows the dominant PC, dashed yellow line shows the sign-flipped PC (after flipping) and the shading represents the 2σ error region propagated from the dating uncertainties for (**c**) PC1 and (**c**) PC2 respectively.

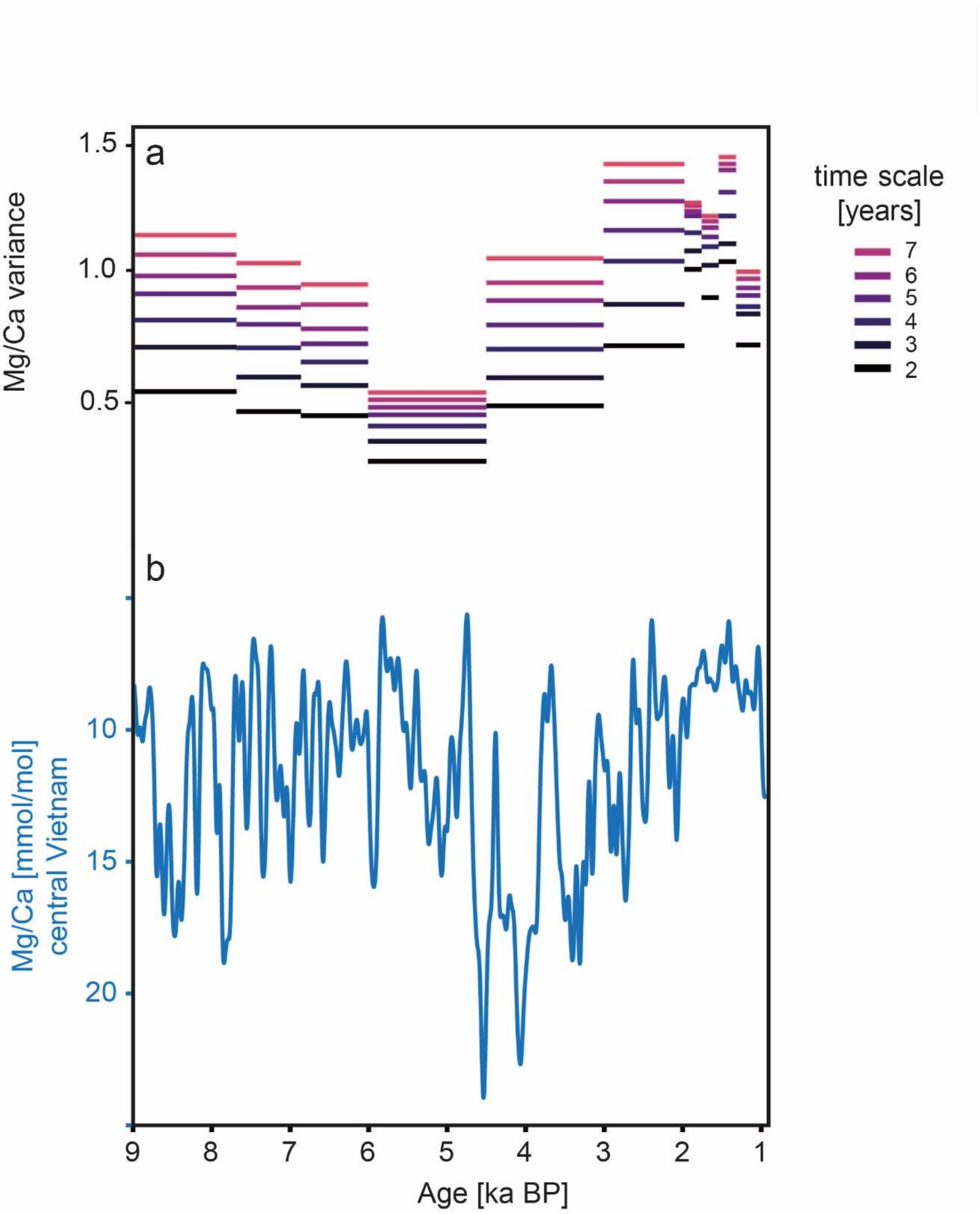

Fig. S8. ENSO band variance in Mg/Ca of TD3.

(a) Normalised Mg/Ca variance of TD3 for the ENSO band (2 to 7 years) and (**b)** the smoothed Mg/Ca records of TD3.

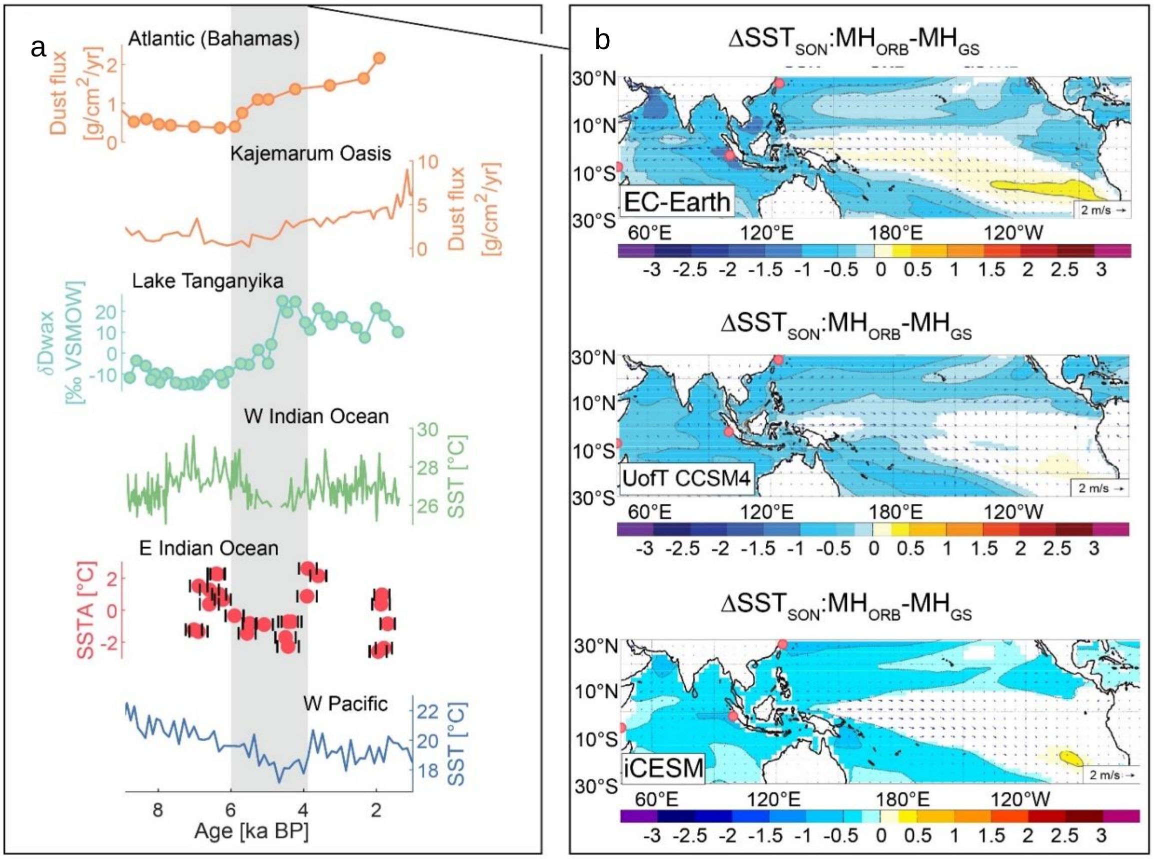

Fig. S9: Sea surface temperatures at the end of the Green Sahara period.

(a) Dust flux over West Africa is recorded in a marine record near the Bahamas[7], and in sediment cores from the Kajemarum Oasis[8] and lake Tanganyika[9]. Annual mean sea surface temperature (SST) based on Mg/Ca from a marine core of the coast of Tanzania (western Indian Ocean)[10], sea surface temperature anomalies (SSTA) reconstructed from corals in the eastern Indian Ocean[11] and SST from South China Sea (western Pacific)[12], pink dots show location of the proxy records. **(b)** Anomalies in SSTS (shading) and surface winds (arrows) as simulated by our three climate models (EC-Earth, UofT CCSM4 and iCESM) between the mid Holocene (6 ka BP) without (MH_{ORB}) and with (MH_{GS}) Saharan vegetation. In particular, the EC-Earth and iCESM changes in airborne dust are accounted for, while in UofT CCSM4 the lake and soil feedback are included. Only changes that are significantly different at the 5% level using a local (gridpoint) *t* test are shaded. The contours follow the color bar intervals (solid for positive and dashed for negative anomalies; the zero line is omitted).

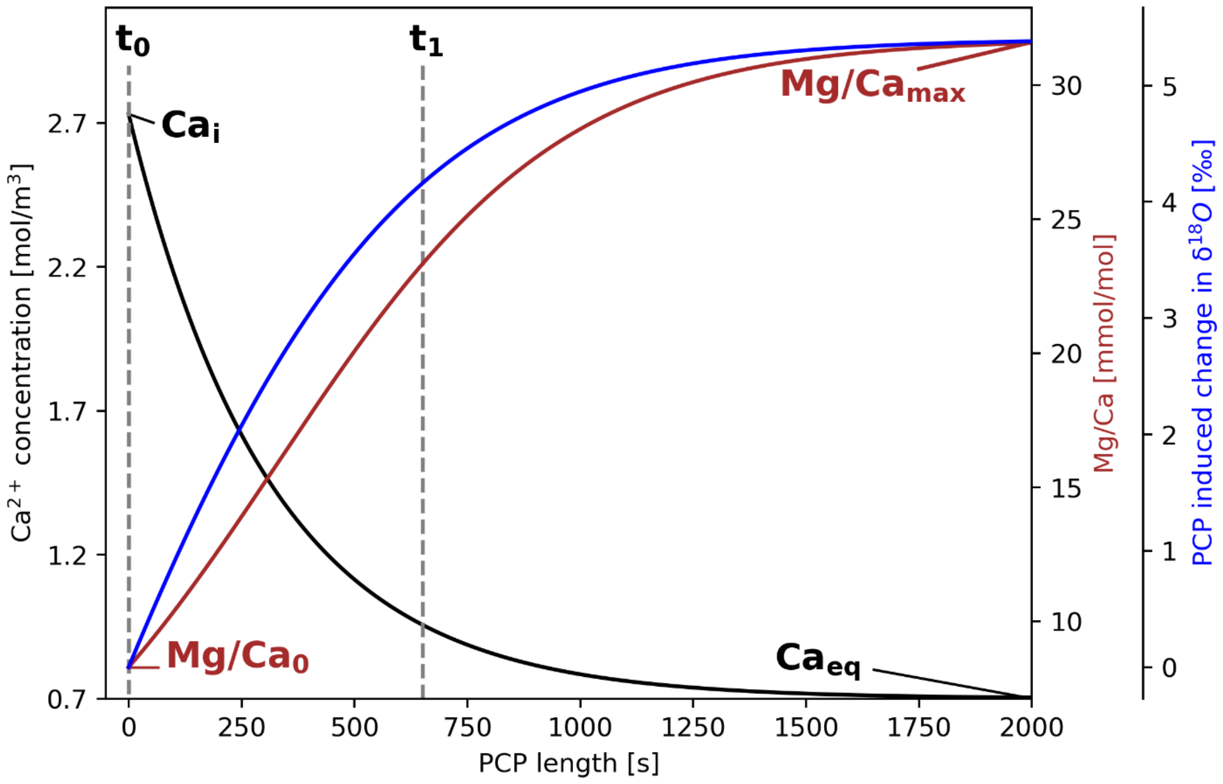

Fig. S10. Simulated calcite precipitation during PCP.

Simulated calcite precipitation during PCP in seconds. Black line shows the exponentially decreasing Ca^{2+} concentration as in Skiba & Fohlmeister, 2023[13] and others[14,15]. Its evolution starts at the initial Ca^{2+} concentration at t_0 (duration of PCP = 0 s) and approximates the Ca^{2+} concentration when then drip water is in equilibrium with cave air pCO_2 (Ca_{eq}) with progressing time. Brown line represents Mg/Ca evolution with the initial Mg/Ca value at t_0 (Mg/Ca_0) approaching the highest Mg/Ca value of the speleothem record (Mg/Ca_{max}) with progressing calcite precipitation. The blue line shows the corresponding PCP-induced change in $\delta^{18}O$. A specific duration of PCP corresponds to a specific change in Mg/Ca and $\delta^{18}O$ (see example for t_1 in the methods section of the main text).

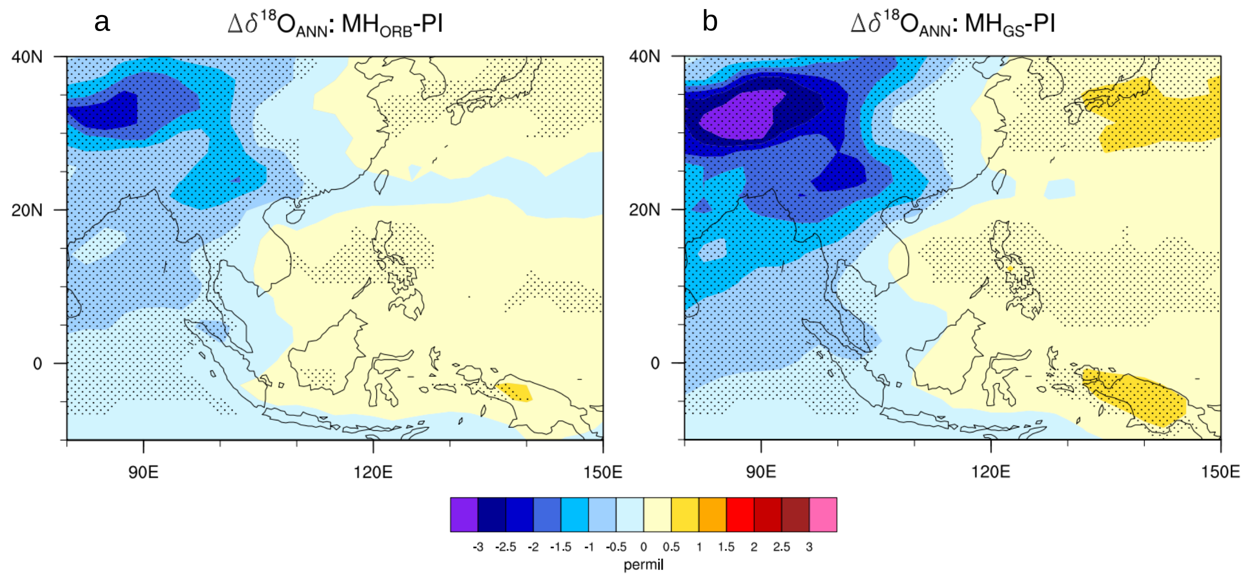

Fig. S11: iCESM simulation. Change in δ^{18}O of annual precipitation for mid-Holocene orbital forcing – preindustrial (PI; 1850 CE) conditions (left, (a)) and mid-Holocene orbital + the Green Sahara forcing – PI (right (b)). Stippling highlight changes significantly different at the 5% level using a *t* test.

Tab. S1: Monte Carlo-PCA EOFs for δ^{18}O records.

EOFs δ^{18}O original					δ^{18}O original	
Sumatra	Vietnam	Borneo	Laos	Dongge	R^2	2σ
2.65E-01	-2.89E-01	-4.79E-01	5.53E-01	5.56E-01	5.62E-01	1.90E-02
7.96E-01	3.62E-01	4.17E-01	6.19E-02	9.97E-02	2.15E-01	2.29E-02
-2.68E-01	8.47E-01	-3.21E-01	2.82E-01	1.39E-02	1.66E-01	1.87E-02

EOFs δ^{18}O corrected					δ^{18}O corrected	
Sumatra	Vietnam	Borneo	Laos	Dongge	R^2	2σ
2.41E-01	-2.51E-01	-4.98E-01	5.77E-01	5.45E-01	5.58E-01	1.90E-02
7.20E-01	5.85E-01	3.15E-01	4.46E-02	1.93E-01	2.59E-01	2.30E-02
-4.69E-01	7.59E-01	-4.26E-01	3.23E-02	1.32E-01	1.21E-01	1.68E-02

EOFs δ^{13}C corrected					δ^{13}C corrected	
Sumatra	Vietnam	Borneo	Laos	Huguang Maar	R^2	2σ
4.63E-01	2.70E-01	4.10E-01	6.16E-01	3.73E-01	3.13E-01	2.65E-02
-5.91E-01	5.73E-01	1.88E-01	-2.21E-01	4.32E-01	2.67E-01	2.23E-02
-1.65E-03	2.49E-01	-8.07E-01	4.32E-01	1.57E-01	1.92E-01	2.25E-02

Supplement references

1. Griffiths, M. L. *et al.* End of Green Sahara amplified mid-to late Holocene megadroughts in mainland Southeast Asia. *Nat. Commun.* **11**, 1–12 (2020).
2. Chawchai, S. *et al.* Hydroclimate variability of central Indo-Pacific region during the Holocene. *Quat. Sci. Rev.* **253**, 106779 (2021).
3. Chen, S. *et al.* A high-resolution speleothem record of western equatorial Pacific rainfall: Implications for Holocene ENSO evolution. *Earth Planet. Sci. Lett.* **442**, 61–71 (2016).
4. Wurtzel, J. B. *et al.* Tropical Indo-Pacific hydroclimate response to North Atlantic forcing during the last deglaciation as recorded by a speleothem from Sumatra, Indonesia. *Earth Planet. Sci. Lett.* **492**, 264–278 (2018).
5. Griffiths, M. L. *et al.* Increasing Australian – Indonesian monsoon rainfall linked to early Holocene sea-level rise. *Nat. Geosci.* **2**, 636–639 (2009).
6. Yancheva, G. *et al.* Influence of the intertropical convergence zone on the East Asian monsoon. *Nature* **445**, 74–77 (2007).
7. Williams, R. H. *et al.* Glacial to Holocene changes in trans-Atlantic Saharan dust transport and dust-climate feedbacks. *Sci. Adv.* **2**, e1600445 (2016).
8. Cockerton, H. E., Holmes, J. A., Street-Perrott, F. A. & Ficken, K. J. Holocene dust records from the West African Sahel and their implications for changes in climate and land surface conditions. *J. Geophys. Res. Atmos.* **119**, 8684–8694 (2014).
9. Tierney, J. E. *et al.* Northern hemisphere controls on tropical southeast African climate during the past 60,000 years. *Science (80-.).* **322**, 252–255 (2008).
10. Kuhnert, H. *et al.* Holocene tropical western Indian Ocean sea surface temperatures in covariation with climatic changes in the Indonesian region. *Paleoceanography* **29**, 423–437 (2014).
11. Abram, N. J., McGregor, H. V, Gagan, M. K., Hantoro, W. S. & Suwargadi, B. W. Oscillations in the southern extent of the Indo-Pacific Warm Pool during the mid-Holocene. *Quat. Sci. Rev.* **28**, 2794–2803 (2009).
12. Rosenthal, Y., Linsley, B. K. & Oppo, D. W. Pacific Ocean heat content during the past 10,000 years. *Science (80-.).* **342**, 617–621 (2013).
13. Skiba, V. & Fohlmeister, J. Contemporaneously growing speleothems and their value to decipher in-cave processes–A modelling approach. *Geochim. Cosmochim. Acta* **348**, 381–396 (2023).
14. Dreybrodt, W. Deposition of calcite from thin films of natural calcareous solutions and the growth of speleothems. *Chem. Geol.* **29**, 89–105 (1980).
15. Fohlmeister, J. *et al.* Main controls on the stable carbon isotope composition of speleothems. *Geochim. Cosmochim. Acta* **279**, 67–87 (2020).
